# Impaired Reality Testing in Mice Lacking Phospholipase Cβ1: Observed by Persistent Representation-Mediated Taste Aversion

**DOI:** 10.1371/journal.pone.0146376

**Published:** 2016-01-05

**Authors:** Hea-jin Kim, Hae-Young Koh

**Affiliations:** 1 Center for Neuroscience, Brain Science Institute, Korea Institute of Science and Technology (KIST), Seoul, 136–791, Republic of Korea; 2 Department of Neuroscience, Korea University of Science and Technology (UST), Daejon, 305–333, Republic of Korea; Mayo Clinic College of Medicine, UNITED STATES

## Abstract

Hallucinations and delusions are the most prominent symptoms of schizophrenia and characterized by impaired reality testing. Representation-mediated taste aversion (RMTA) has been proposed as a potential behavioral assessment of reality testing and has been applied to a neurodevelopmental rat model of schizophrenia. However, the theory underlying this approach has not been generalized yet with any demonstration of impaired reality testing in other animal models of schizophrenia, such as genetically-modified mice. We devised a RMTA procedure for mice that combines a Pavlovian association protocol pairing odor conditioned stimulus (CS) with sugar reward unconditioned stimulus (US), and a conditioned taste aversion (CTA) method. In this RMTA paradigm, we compared performances of wild-type (PLCβ1^+/+^) mice and phospholipase C β1 knock-out (PLCβ1^-/-^) mice which are known as one of the genetic models for schizophrenia. With a minimal amount of initial odor-sugar associative training, both PLCβ1^+/+^ and PLCβ1^-/-^ mice were able to form an aversion to the sugar reward when the odor CS predicting sugar was paired with nausea. With an extended initial training, however, only PLCβ1^-/-^ mice could form a RMTA. This persistent RMTA displayed by PLCβ1^-/-^ mice shows their inability to distinguish real sugar from the CS-evoked representation of sugar at a stage in associative learning where wild-type mice normally could differentiate the two. These results demonstrate an impaired reality testing first observed in a genetic mouse model of schizophrenia, and suggest that RMTA paradigm may, with general applicability, allow diverse biological approaches to impaired reality testing.

## Introduction

Hallucinations are the most typical and debilitating of the core symptoms of schizophrenia. They are encountered also in mood disorders [1], Parkinson's disease [2], Alzheimer’s disease [3], and even in a small proportion of general population [4]. Due to the limited view that hallucination is an uniquely human pathological phenomenon, most researches have been carried out mainly by functional brain imaging studies (e.g., fMRI) on human patients [2, 5–7], with its underlying neurobiology remaining elusive. Physiological research on behavior related to major symptoms of mental illnesses requires phylogenetically-relevant animal model behaviors [8, 9]. Considered in ethological term, hallucinations are characterized by impaired reality testing. Like in other relatively successful animal models, impaired reality testing could be seen as a condition that involves an otherwise normal physiological phenomenon occurring yet in aberrant contexts.

Impaired reality testing is defined as inability to distinguish between actual event and internal representation of an absent event. And this kind of state can be observed by animal behaviors in some of the normal phenomena in Pavlovian associative learning. In associative learning, a conditioned stimulus (CS) comes to activate an internal representation of the unconditioned stimulus (US) with which it is paired, and this representation can serve for acquisition of new learning about US. For instance, rats that have received tone-sugar (CS-US) pairings, later learn to reduce consumption of sugar reward when they are presented with the auditory CS alone paired with LiCl-induced nausea. Thus, CS-evoked representation of a food US could substitute for the actual US itself in the acquisition of an aversion to that US, which is called representation-mediated taste aversion (RMTA) [10, 11]. Normally, RMTA occurs only transiently in the early stage of the initial Pavlovian conditioning, and CS→nausea pairing can no longer establish an aversion to the US later with an extended initial conditioning, so that the sensitivity to RMTA changes as training proceeds [12, 13]. It was proposed that, with a minimal amount of training, CS evokes a highly realistic, sensory representation of the food US that is not fully distinguished from the actual US, like a hallucination in that animals actually “taste” the food while exposed to CS, and that, with an extended training, this sensory representation is replaced by a less perceptual one that is distinguishable from the actual US [11, 14]. Based on this learning theory, McDannald and Schoenbaum proposed to apply RMTA procedures to animal models for impaired reality testing, expecting to find cases in which the normal course of sensitivity to RMTA is disrupted [15]. RMTA has been applied to a neurodevelopmental animal model of schizophrenia: the rats given neonatal ventral hippocampal lesions (NVHL), which demonstrated an overall enhanced RMTA compared with control rats [16]. However, impaired reality testing demonstrated via RMTA paradigm has not been established yet in other animal models of schizophrenia, such as genetically-modified mice.

Using a RMTA procedure with odor-food association devised for mice, here we have compared the effects of amount of initial training in wild-type mice and in the phospholipase C β1 knockout mice (PLCβ1^-/-^) [17]. PLCβ1^-/-^ mice are known to be one of the genetic mouse models for schizophrenia [18], displaying relevant behavioral phenotypes including, deficit in sensorimotor gating, hyperactivity, abnormal social behaviors, and impaired working memory [19, 20]. Even though the performance of PLCβ1^-/-^ mice in some learning tasks (fear conditioning, Morris water maze tests) is known to be somewhat questionable [21, 22], the present study has shown that PLCβ1^-/-^ mice are as competent at both olfactory discrimination and conditioned taste aversion learning as wild-type, which allows us to design a RMTA procedure using odor-food association. By detecting an apparent effect of amount of training on the occurrence of RMTA in wild-type mice, and a persistent RMTA in PLCβ1^-/-^ mice, we could observe both the normal and aberrant courses of sensitivity to RMTA in mice. Allowing a variety of molecular and physiological assessments, such as attempting to determine the role of PLCβ1 in the normal transition in the nature of CS-evoked US representation, the study with PLCβ1^-/-^ mice using RMTA paradigm will contribute to biological understanding of impaired reality testing.

## Materials and Methods

### Subjects

Phospholipase Cβ1 (PLCβ1) wild-type and mutant mice were obtained by crossing C57BL/6J (N28) PLCβ1^+/-^ and 129S4/SvJae (N39) PLCβ1^+/-^ mice as described previously [17]. Subjects were male mice ~15 weeks old at the start of the experiment. Mice were deprived of food for 24 hours before the start of the experiment. Throughout the study, weight was monitored daily and was maintained by supplemental feedings of 3.5-g standard lab diet chow 1–3 hours after the last experimental session of the day. As a result, the weight during the experiment was reduced to 85–90% of free-feeding body weight, the mean weight for 3 days prior to food restriction. Subjects were housed individually in home cage and were maintained on a 12:12 –light/dark schedule with light on at 08:30 AM. Behavioral experiments were generally conducted between 10:00 AM and 4:00 PM and each animal was used only for one experiment. All experiment including animal care and handling procedures were performed in accordance with the institutional guidelines and regulations of Korea Institute of Science and Technology (KIST). All experimental protocols were approved by the institutional Animal Care and Use Committee (IACUC) of KIST (AP201149).

### 1. Olfactory Discrimination

#### 1.1. Apparatus

**Fig 1:**
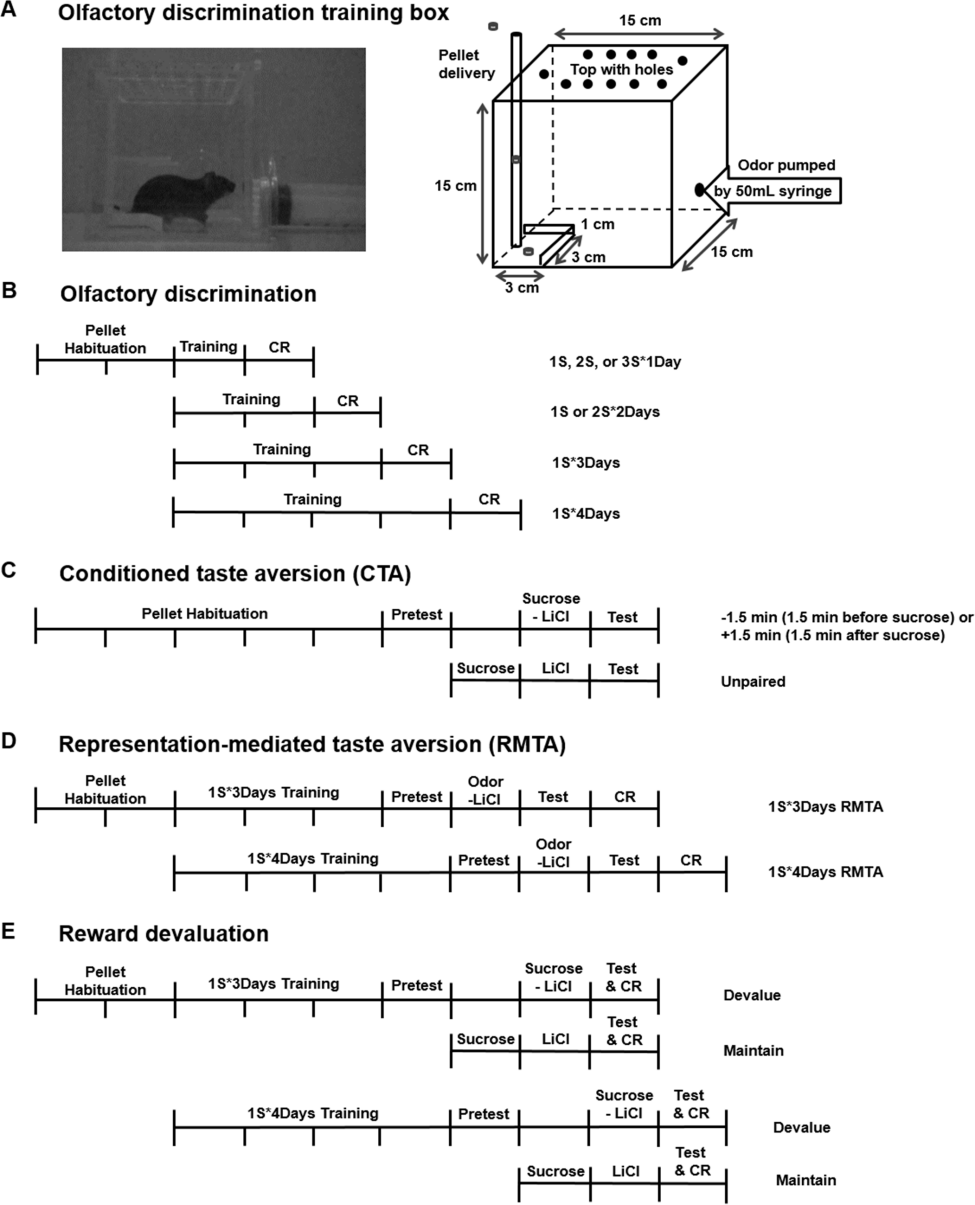
Apparatus for olfactory discrimination and experimental outlines. (A) Olfactory discrimination training and test were conducted in a clear acyl box. (B) Olfactory discrimination took place over 4 to 7 days. After 2 days of habituation to food pellets, olfactory training sessions (+odor→sugar pairing, -odor without sugar) were given in varying numbers of sessions per day and of training days. Conditioned behavioral responding (CR) to +odor was tested on the CR day. (C) Conditioned taste aversion (CTA) took place over 9 days. After 5 days of habituation, a baseline sugar consumption test (Pretest) was given on Day 6. Paired mice were given LiCl injection 1.5 min before (-1.5 min) or after (+1.5 min) access to sugar pellets on Day 8, whereas Unpaired control mice were given sugar and nausea separately on Days 7 and 8. On Day 9, a post-nausea sugar consumption test (Test) was given. (D) Representation-mediated taste aversion took place over 9 or 10 days. After 2 days of habituation, +odor→sugar conditioning was given for either 3 (1S*3Days) or 4 days (1S*4Days). On Day 6/7, a baseline sugar consumption test was given. On Day 7/8, odor→nausea pairing was given, with half the mice receiving +odor and half receiving -odor. A post-nausea sugar consumption test was given on 8/9, and CR was tested on Day 9/10. (E) Reward devaluation took place over 9 or 10 days. After 2 days of habituation, +odor→sugar conditioning was given for either 3 (1S*3Days) or 4 days (1S*4Days), and CR was measured on the last day (pre-nausea CR). On Day 6/7, a baseline sugar consumption test was given. On Day 8/9, Devalue mice were given sugar→nausea pairing, and Maintain mice were given sugar and nausea separately on Days 7/8 and 8/9. Post-nausea sugar consumption and post-nausea CR were tested on Day 9/10.

Olfactory discrimination was conducted in a 15 X 15 X 15 cm acyl box (Fig 1A). This box had a hole at the center of the front wall 2 cm high from the bottom to be connected to a syringe for odor presentation, and a top plate with many holes for odor ventilation. Cocoa (Hershey’s Cocoa–unsweetened 1 g,) and cinnamon (ISFI Cinnamon Powder 0.5 g) were used as a food-paired (+odor) or a food-unpaired odor (-odor). For odor presentation, saturated odor was pumped in through the front center hole to the acryl box using 50-ml syringe (Kovax Syringe). A square food cup (3 X 3 X 1 cm) was located at the left bottom corner of the back wall. The food cup was connected to a tube feeder mounted from the outside of the top plate to deliver 20-mg sucrose pellets (Dustless Precision Pellets, Rodent Purified Diet, Bio Serve, Flemington, New Jersey).

#### 1.2. Procedure

**Fig 2:**
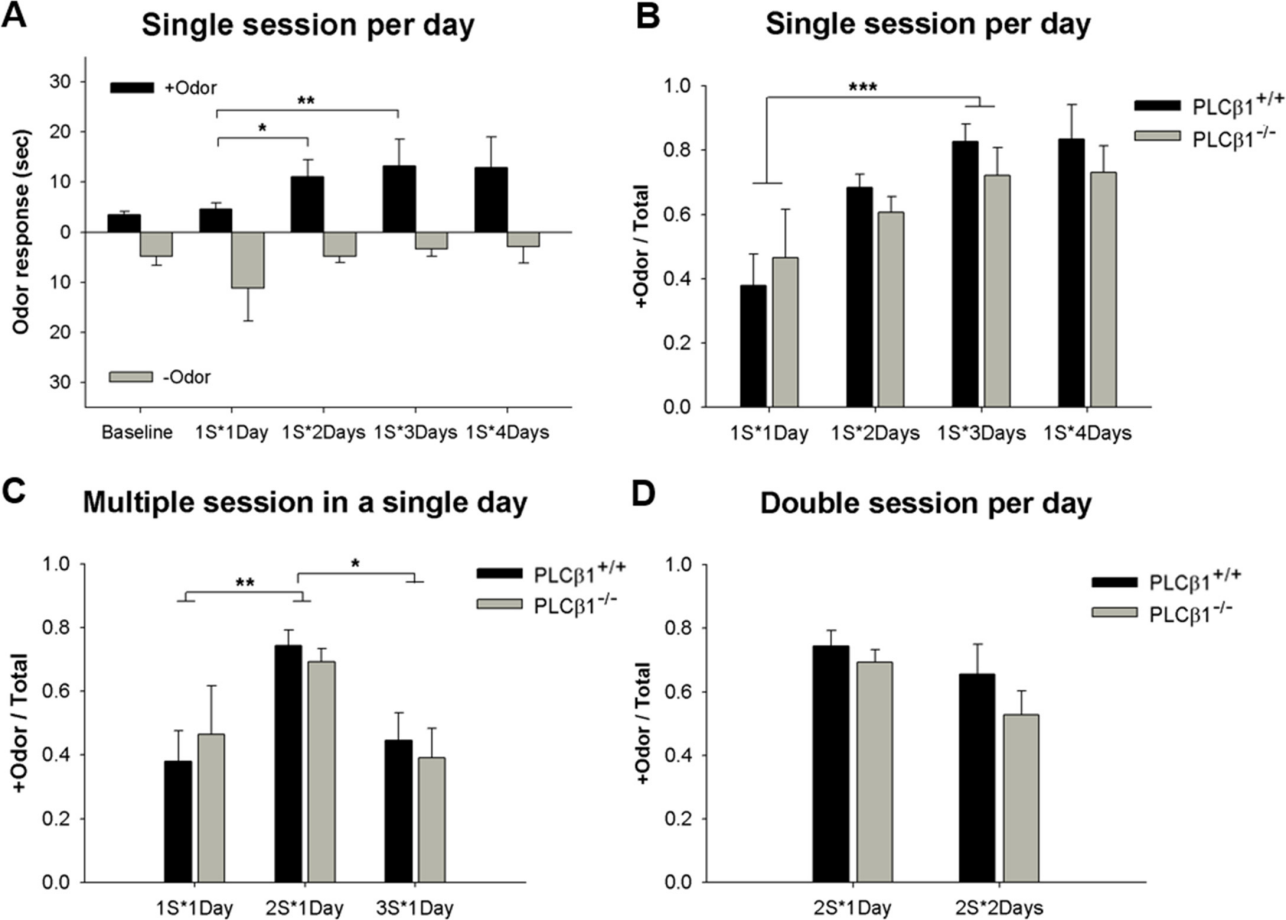
Effect of numbers of training days and sessions/day in olfactory discrimination performance of PLCβ1^+/+^ and PLCβ1^-/-^ mice. (A) Absolute time spent in the food cup in response to the odor that has been paired with sucrose pellets (+odor, black) and the one not paired with sucrose (-odor, gray) without any training (Baseline) were 4.3 ± 0.7 and 5.5 ± 1.8 sec, respectively (n = 8: PLCβ1^+/+^, n = 5; PLCβ1^-/-^, n = 3). +odor response increased with increasing number of training days (1S*2Days, *p* = .04; 1S*3Days, *p* = .000; compared to 1S*1Days) but -odor response did not change significantly (*p* > .05), both genotypes included. (B) Relative time spent seeking food in response to +odor (+Odor / Total) with increasing number of training days: one training session per day, 1 to 4 days (1S*1Days ~ 1S*4Days), for PLCβ1^+/+^ (black) and PLCβ1^-/-^ mice (gray). (C) Learning with increasing number of sessions/day: one training day, 1 to 3 sessions. PLCβ1^+/+^ (black), PLCβ1^-/-^ (gray). (D) Learning with one or two double-session days. PLCβ1^+/+^ (black), PLCβ1^-/-^ (gray). All values are Mean ± SEM. *p < .05; **p < .01; ***p < .001.

The experimental timeline is shown in Fig 1B. Before experiment, all subjects (PLCβ1^+/+^, n = 58; PLCβ1^-/-^, n = 55) were habituated to the acyl box and food pellets (Grain-based Pellets, Rodent Purified Diet, Bio Serve, Flemington, New Jersey) for 2 days. The following day, all subjects were given the opportunity to explore the acryl box freely for 3 min before the odor presentation. One odor (+odor) was paired with sucrose pellets (3 pellets) by dropping them through the tube feeder into the food cup about 5 sec after the odor pumping. About 90 sec after the odor pumping, subjects were returned to the home-cage. Approximately 20 min later, subjects received another trial with the other odor (-odor) that was not paired with pellets (counterbalanced). These two trials, one for +odor and another for -odor, made up one session. This procedure is modified from the methods by Schellinck [23]. Mice were trained with varying number of sessions per day for varying number of days (Table 1). The sequence of +odor and -odor trials was made random in each schedule. Test session was performed on the day following the last session. This session was similar to the training session but did not provide sugar pellets. The time spent seeking sugar pellets in the food cup in response to each odor during the 90-sec period after the odor pumping was measured by analyzing video records. Conditioned response (CR), or the measure of association between +odor and sugar pellets, was the relative time spent in food cup in response to +odor: +odor / total = [time in food cup in response to +odor / (time in food cup in response to -odor + time in food cup in response to +odor)]. The absolute values of time spent in the food cup in response to +odor and -odor without any training (baseline) and with increasing number of training days are shown in Fig 2A.

**Table 1:** Olfactory discrimination learning schedules for varying amount of training.

Number of sessions/day	1 day	2 days	3 days	4 days
**1 session**	1S*1Day	1S*2Days	1S*3Days	1S*4Days
**2 sessions**	2S*1Day	2S*2Days		
**3 sessions**	3S*1Day			

Symbols for combinations of the number of training sessions per day (x) and the number of training days (y) are shown as xS*yDays (e.g. 2S*2Days = 2 sessions per day for 2 days). Mice of each genotype group were divided into 7 groups: 1S*1Day (PLCβ1^+/+^, n = 8; PLCβ1^-/-^, n = 5), 2S*1Day (PLCβ1^+/+^, n = 11; PLCβ1^-/-^, n = 11), 3S*1Day (PLCβ1^+/+^, n = 9; PLCβ1^-/-^, n = 10), 1S*2Days (PLCβ1^+/+^, n = 10; PLCβ1^-/-^, n = 5), 2S*2Days (PLCβ1^+/+^, n = 5; PLCβ1^-/-^, n = 6), 1S*3Days (PLCβ1^+/+^, n = 7; PLCβ1^-/-^, n = 12), 1S*4Days (PLCβ1^+/+^, n = 8; PLCβ1^-/-^, n = 6).

### 2. Conditioned Taste Aversion

Mice were kept in their home-cages during the conditioned taste aversion (CTA) experiment. The experimental timeline is shown in Fig 1C. All subjects (PLCβ1^+/+^, n = 30; PLCβ1^-/-^, n = 21) were habituated for 5 days to sucrose pellets and food cup, by giving them 60 pellets for 1 hour. On day 6 and 9, pretest and test trials assessed the amount of sucrose pellets consumed in 20 min. On day 7, mice of each genotype group were divided into 3 groups based on the timing between sucrose presentation and LiCl injection, either paired or unpaired: -1.5 min, +1.5 min, Unpaired, and only Unpaired group (PLCβ1^+/+^, n = 8; PLCβ1^-/-^, n = 6) was given access to sugar pellets. On day 8, -1.5 min group (PLCβ1^+/+^, n = 11; PLCβ1^-/-^, n = 9) was injected intraperitoneally with lithium chloride (LiCl, 0.15 M, 30 mL/kg) (Sigma-Aldrich, St. Louis, MO) 1.5 min before the start of access to sucrose pellet, +1.5 min group (PLCβ1^+/+^, n = 11; PLCβ1^-/-^, n = 6) 1.5 min after the end of access to pellets, and Unpaired group was injected with LiCl with no additional exposure to pellets (consequentially, Unpaired group received sugar and nausea 24 hours apart). To be included in consumption analysis for CTA, mice had to eat at least 40 of 60 pellets in the baseline consumption test. The measure of taste aversion was aversion index (AI) = [(number of pellets consumed in pretest trial—number of pellets consumed in test trial) / number of pellets consumed in pretest trial]. Positive numbers indicate that a taste aversion was formed.

### 3. Representation-Mediated Taste Aversion

Representation-mediated taste aversion (RMTA) paradigm consisted of an olfactory discrimination learning and a conditioned taste aversion (CTA) paradigm that pairs +odor with LiCl (Fig 1D). This procedure is modified from the method by Saddoris and Wheeler [24, 25]. All subjects (PLCβ1^+/+^, n = 38; PLCβ1^-/-^, n = 38) were single-housed and habituated to pellets by giving them 60 grain-based pellets for 1 hour every day for 2 days. Subjects of each genotype were divided into 1S*3Days (PLCβ1^+/+^, n = 17; PLCβ1^-/-^, n = 15) and 1S*4Days (PLCβ1^+/+^, n = 21; PLCβ1^-/-^, n = 23) groups for olfactory learning. 1S*3Days group was given a single olfactory discrimination training session per day for 3 days, and 1S*4Days group for 4 days, as described in “olfactory discrimination” procedure section. In pretest trial of the CTA paradigm, all subjects were given 60 pellets and the number of pellets consumed in 20 min was counted in home-cage. The next day, mice of each training group were divided into experiment (Exp) (1S*3Days: PLCβ1^+/+^, n = 9; PLCβ1^-/-^, n = 7) (1S*4Days: PLCβ1^+/+^, n = 11; PLCβ1^-/-^, n = 13) and Control (1S*3Days: PLCβ1^+/+^, n = 8; PLCβ1^-/-^, n = 8) (1S*4Days: PLCβ1^+/+^, n = 10; PLCβ1^-/-^, n = 10) groups. Exp group received LiCl (0.15 M, 30 mL/kg) injection 1.5 min before the presentation of +odor in the olfactory training box. Control group received LiCl (0.15 M, 30 mL/kg) injection 1.5 min before the presentation of -odor instead. In test trial, the number of pellets consumed in 20 min was counted. The degree of mediated taste aversion learning was measured in aversion index (AI) as described in “CTA” procedure section. The following day, conditioned response (CR) for the initial olfactory training was measured, as described in “olfactory discrimination” procedure section.

### 4. Reward Devaluation

The experimental timeline is shown in Fig 1E. Reward devaluation paradigm consisted of an olfactory discrimination learning and a conditioned taste aversion (CTA) paradigm. Before conditioning, all subjects (PLCβ1^+/+^, n = 37; PLCβ1^-/-^, n = 23) were single-housed and habituated to pellets for 1 hour a day for 2 days, as described in the RMTA procedure section. Subjects of each genotype were divided into 1S*3Days (PLCβ1^+/+^, n = 17; PLCβ1^-/-^, n = 12) and 1S*4Days (PLCβ1^+/+^, n = 20; PLCβ1^-/-^, n = 11) groups for olfactory learning. 1S*3Days group was given a single olfactory discrimination training session per day for 3 days, and 1S*4Days group for 4 days, as described in “olfactory discrimination” procedure section. On the last day of olfactory discrimination training, durations of time spent in the food cup for the 30 sec after the odor presentation were recorded to measure the Pre-nausea conditioned response (CR) (“olfactory discrimination” procedure section). In pretest trial of CTA, all subjects were given 60 pellets and the number of pellets consumed in 20 min was counted in home-cage. The next day, mice of each training group were divided into Devalue (1S*3Days: PLCβ1^+/+^, n = 7; PLCβ1^-/-^, n = 5) (1S*4Days: PLCβ1^+/+^, n = 12; PLCβ1^-/-^, n = 6) and Maintain (1S*3Days: PLCβ1^+/+^, n = 10; PLCβ1^-/-^, n = 7) (1S*4Days: PLCβ1^+/+^, n = 8; PLCβ1^-/-^, n = 5) groups, and only Maintain group was given access to sugar pellets. The following day, Devalue group received LiCl injection 1.5 min before the presentation of sugar pellets, and Maintain group received LiCl injection with no additional exposure to sugar (consequentially, Maintain group received sugar and nausea 24 hours apart). In test trial, number of pellets consumed in 20 min was counted. 4 hours later, Post-nausea CR for the initial olfactory learning was measured.

### Statistical Analyses

Data were acquired using SPSS 16.0 software and analyzed with analysis of variance (ANOVA). Post hoc comparisons were made with Tukey’s honestly significant difference. All data are expressed as mean ± SEM. *p* values < .05 were considered statistically significant.

## Results

### 1. Olfactory Discrimination

To be able to examine the effect of amount of initial training on representation-mediated taste aversion (RMTA) learning, it is required to use a CS-US associative learning paradigm where the minimal- and the extended training conditions are decided relatively easily [13]. We used an olfactory discrimination paradigm as an initial conditioning for the RMTA test. In olfactory discrimination, mice were trained to distinguish between the odor paired with sugar pellet reward (+odor) and the unpaired one (-odor) so that +odor predicts sugar pellets. In order to decide the conditions for minimal- and extended training, effects of the numbers of training days and sessions per day on learning were examined. Experimental timeline and training procedures are shown in Fig 1B. In single-session-per-day paradigms, both genotypes showed an increase in conditioned responding (CR, relative time spent seeking sugar in response to +odor) across numbers of training days (Fig 2B). CR was subjected to a two-way ANOVA with factors of genotype (PLCβ1^+/+^, PLCβ1^-/-^) and number of training days (1S*1Day, 1S*2Days, 1S*3Days and 1S*4Days). ANOVA showed a significant main effect of number of training days [F(3,53) = 23.862, *p* = .000], but no significant effect [F(1,53) = 0.04, *p* = .85] of or interaction [F(3, 53) = 0.29, *p* = .83] with genotype. Post hoc comparisons showed that both 1S*3Days and 1S*4Days groups were significantly different from 1S*1Day group in CR (*p*s < .001). The efficiency of learning reached the plateau at 3 days of training (Fig 2B, 1S*3Days). In addition, multiple-session-per-day paradigms were also examined (Fig 2C and 2D). 2S*1Day training was more effective than 1S*1Day (*p*s *<* .01) in both genotypes (Fig 2C). However, 3S*1Day training was not any better than 2S*1Day but even worse (*p*s *<* .05) (Fig 2C). 2S*2Days training was not more efficient than either 2S*1Day training (*p*s *>* .05) (Fig 2D) or 1S*2Days (*p*s *>* .05). Based on these results, 1S*3Days and 1S*4Days were decided as the minimal and the extended training conditions, respectively, for RMTA test.

### 2. Conditioned Taste Aversion: Effect of temporal arrangement of sucrose pellet and LiCl injection

**Fig 3:**
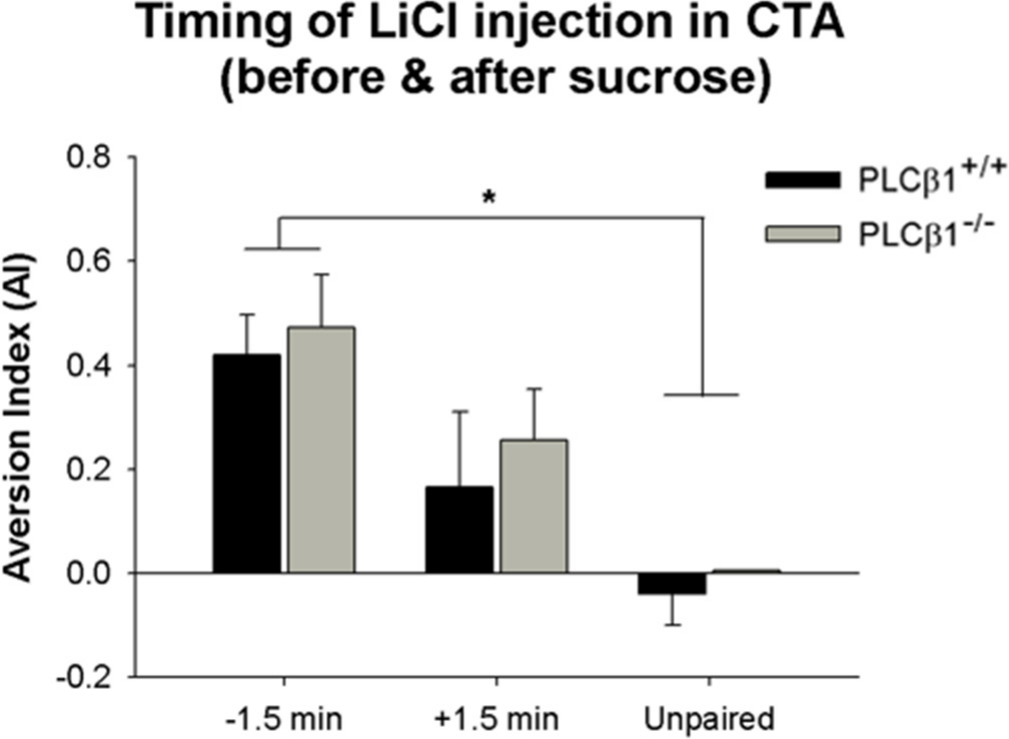
Effect of temporal arrangement of access to sucrose pellets and LiCl injection in conditioned taste aversion learning of PLCβ1^+/+^ and PLCβ1^-/-^ mice. Mean ± SEM aversion index (AI) values for LiCl injections 1.5 minutes before (-1.5 min) and after (+1.5 min) access to sucrose pellets, and for unpaired injection (Unpaired), are shown for PLCβ1^+/+^ (black) and PLCβ1^-/-^ mice (gray). The more positive the value, the greater the taste aversion that was formed. *p < .05.

When animals learn to associate events, the timing of the events is critical [26]. In conditioned taste aversion (CTA), appropriate temporal relationship between the exposure to food flavor and the experience of LiCl-induced illness should be determined for effective learning to occur in specific experimental settings [27]. It had been observed that it takes a few minutes for symptoms of LiCl-induced nausea to come into action in the mice strains used in this study. Therefore, aversion learning could be more readily achieved when mice were injected with LiCl minutes before the actual exposure to food flavor than when injected after. To verify this possibility, the influence on aversion learning of temporal arrangement of food and illness in both genotypes, subjects were injected with LiCl either 1.5 min before the start of (-1.5 min) or 1.5 minutes after the end of (+1.5 min) access to sucrose pellets. Unpaired control subjects (Unpaired) were given sucrose pellets and LiCl injection 24 hours apart. Experimental timeline and training procedures are shown in Fig 1C. PLCβ1^+/+^ and PLCβ1^-/-^ both established an aversion to sucrose pellets when they were injected with LiCl 1.5 min before exposure to sucrose (Fig 3). ANOVA for aversion index (AI) [(genotype—PLCβ1^+/+^, PLCβ1^-/-^) X (group—−1.5 min, +1.5 min, Unpaired)] revealed a significant main effect of group [F(2,45) = 9.39, *p* = .000], but no effect [F(1,45) = 0.45, *p* = .51] of or interaction [F(2,45) = 0.03, *p* = .97] with genotype. Post hoc comparisons found significant differences between -1.5 min and Unpaired groups in AI in both genotypes (*p*s < .05). Based on these results, the protocol of injecting LiCl 1.5 min before stimulus (exposure to +odor or sucrose pellets) was used later in both representation-mediated taste aversion and reward devaluation experiments.

### 3. Representation-Mediated Taste Aversion: Effects of amount of initial training

**Fig 4:**
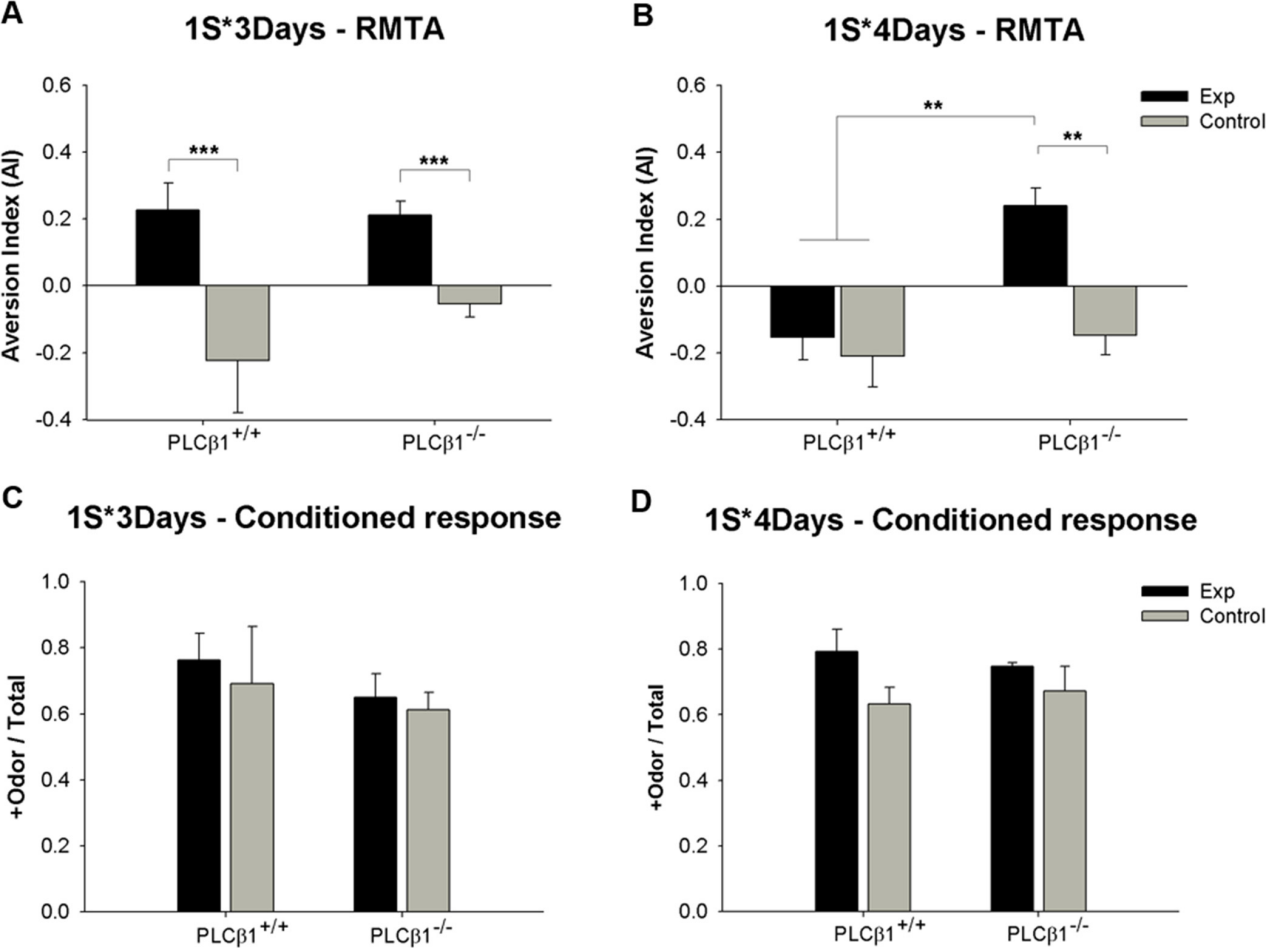
Representation-mediated taste aversion (RMTA) performance of PLCβ1^+/+^ and PLCβ1^-/-^ mice with minimal (1S*3Days) and extended (1S*4Days) olfactory discrimination trainings. (A), (B) Aversion index (AI) values for PLCβ1^+/+^ and PLCβ1^-/-^ mice that were given +odor→nausea (Exp, black) or -odor→nausea pairing (Control, gray) after 3 days (1S*3Days) or 4 days (1S*4Days) of olfactory discrimination training. (C), (D) Conditioned response tested after RMTA test for the same groups shown in (A) and (B). Conditioned response is the relative time spent seeking food in response to +odor (+Odor / Total). All values are Mean ± SEM. **p < .01; ***p < .001.

Effects of amount of initial training on representation-mediated taste aversion (RMTA) learning were examined in PLCβ1^+/+^ and PLCβ1^-/-^ mice, using the optimal conditions and protocols for olfactory discrimination and conditioned taste aversion (CTA). Experimental timeline and training procedures are shown in Fig 1D. PLCβ1^+/+^ and PLCβ1^-/-^ mice were trained for either 3 days or 4 days (1S*3Days, 1S*4Days) for olfactory discrimination as an initial conditioning that +odor predicted sucrose pellets. Mice in experiment (Exp) group were injected with LiCl 1.5 minutes before exposure to +odor without sucrose pellets present (+odor→nausea pairing). Control group received a pairing of -odor and LiCl-induced nausea (-odor→nausea). In 1S*3Days case, both the PLCβ1^+/+^ and PLCβ1^-/-^ mice succeeded in mediated taste aversion (Fig 4A). When compared with control groups, both PLCβ1^+/+^ and PLCβ1^-/-^ Exp groups formed a significant aversion to sucrose pellets. ANOVA [(genotype—PLCβ1^+/+^, PLCβ1^-/-^) X (group—Exp, Control)] for aversion index [AI = (number of pellets consumed in pretest trial—number of pellets consumed in test trial) / number of pellets consumed in pretest trial] found a main effect of group [*F*(1,28) = 85.248, *p* = .000] but no effect of genotype [*F*(1,28) = 4.05, *p* = .05]. There was a significant genotype X group interaction [*F*(1,28) = 5.68, *p* = .024] due to a significantly more negative AI of PLCβ1^+/+^ compared to PLCβ1^-/-^ in Control groups (*p* = 0.02). Post hoc comparisons found significant differences between Exp and Control AI values in both genotypes (*p*s = .000). Subsequently, a conditioned responding (CR, relative time spent seeking sugar in response to +odor) test was conducted in which odors were presented in the training chamber without sucrose pellets present (Fig 4C). Mice in all groups showed sucrose-seeking behavior in response to +odor. ANOVA [(genotype—PLCβ1^+/+^, PLCβ1^-/-^) X (group—Exp, Control)] for CR revealed no significant main effect or interaction (*p*s > .1). Since the presentations of odor CS and sugar US could be partially overlapped during +odor→sugar pairing in the initial olfactory discrimination training, one might concern that sugar→+odor (backward) association could happen as well as +odor→sugar, and thus, at test, the taste of sugar pellet could be evoking a representation of +odor, and in turn the reduction in sugar consumption could be due to aversive conditioning to +odor itself, which was directly paired with nausea. However, the fact that +odor’s ability to evoke conditioned response was not affected by the +odor→nausea pairing (Fig 4C), makes the aforementioned case improbable.

In 1S*4Days case, however, only PLCβ1^-/-^ Exp group showed a significant aversion to sucrose pellets (Fig 4B) compared with other groups. ANOVA [(genotype—PLCβ1^+/+^, PLCβ1^-/-^) X (group—Exp, Control)] for AI found main effects of genotype [*F*(1,40) = 8.93, *p* = .005] and group [*F*(1,40) = 12.96, *p* = .001], and a significant genotype X group interaction [*F*(1,40) = 4.21, *p* = .047]. Post hoc comparisons found a significant difference in AI between Exp and Control groups of PLCβ1^-/-^ (*p* = .001), and also a significant difference between both groups of PLCβ1^+/+^ and Exp group of PLCβ1^-/-^ (*p*s < .003). On the contrary, there was no difference in CR among groups (Fig 4D). ANOVA [(genotype—PLCβ1^+/+^, PLCβ1^-/-^) X (group—Exp, Control)] for CR revealed no significant main effect or interaction (*p*s > .05). These results suggest that, with 4 days of initial conditioning (extended training), PLCβ1^-/-^, but not PLCβ1^+/+^, mice formed a RMTA, and that the inability of PLCβ1^+/+^ mice to perform mediated learning was not due to a loss of the association between +odor and sugar reward. ANOVA for AI which included training amount (1S*3Days, 1S*4Days) as a factor found a significant interaction among the three factors [*F*(1,68) = 7.259, *p* = .009], but no effect or interaction for CR (*p*s > .05).

### 4. Reward Devaluation

Theoretically, RMTA depends on the type of initial conditioning being a stimulus-stimulus (S-S) learning where CS evokes internal representation of US reward. In case that the initial conditioning is established in a stimulus-response (S-R) type where CR is performed without mediation by US representation, RMTA cannot occur [11, 14]. Even though it was shown that PLCβ1^+/+^ still maintained associative learning between odor CS and sugar reward with 4 days of training (Fig 4D), there is also a possibility that the conditioned behavioral response observed in this occasion could be the result of a S-R learning due to automatization of the learned behavior after an extended training, rather than of the S-S learning. To examine this possibility, reward devaluation tests were performed [12, 14, 28]. In reward devaluation task, the conditioned animals are given sugar→nausea pairing (direct taste aversion training), and then tested for their behavioral respondings to the CS, to decide whether the conditioned responding is affected by the current value of the sugar reward. If the observed conditioned responding is the result of S-S, and not S-R learning, it should be reduced after the animals have once learned about the lowered value of the sugar reward.

**Fig 5:**
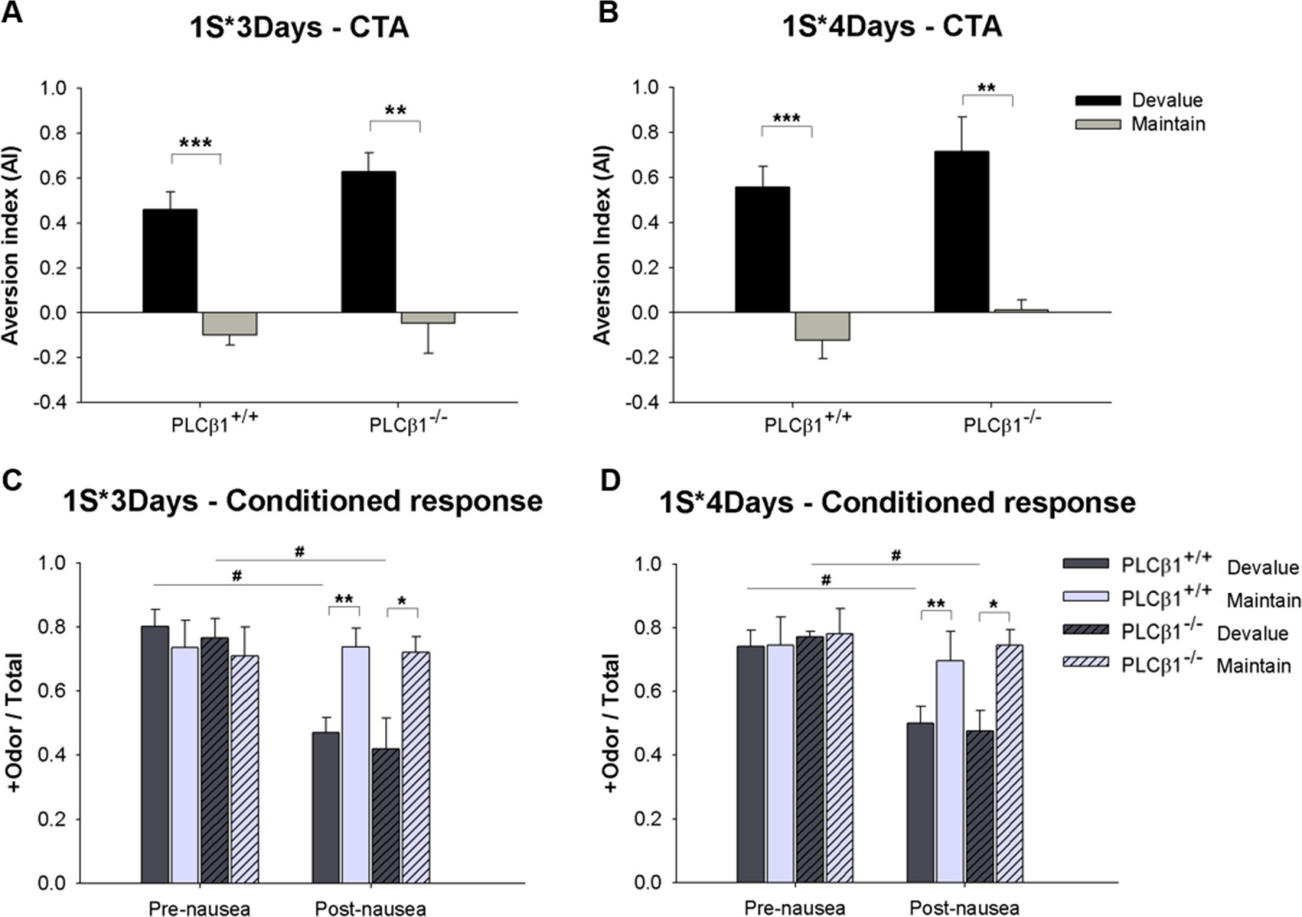
Reward devaluation performance of PLCβ1^+/+^ and PLCβ1^-/-^ mice with minimal (1S*3Days) and extended (1S*4Days) olfactory discrimination trainings. (A), (B) Aversion index (AI) values for PLCβ1^+/+^ and PLCβ1^-/-^ mice that were given sugar→nausea pairing (Devalue, black) or unpaired sugar and nausea (Maintain, gray) after 3 days (1S*3Days) or 4 days (1S*4Days) of olfactory discrimination training. (C), (D) Conditioned response tested before (Pre-nausea) and after (Post-nausea) the CTA procedure for the same groups shown in (A) and (B). PLCβ1^+/+^ (plain), PLCβ1^-/-^ (hatched), Devalue (dark), Maintain (light). Conditioned response is the relative time spent seeking food in response to +odor (+Odor / Total). All values are Mean ± SEM. *p < .05; **p < .01; ***p < .001; ^#^*p* < .05.

Reward devaluation test was performed in PLCβ1^+/+^ and PLCβ1^-/-^ mice, using the optimal conditions and protocols for olfactory discrimination and conditioned taste aversion (CTA). Experimental timeline and training procedures are shown in Fig 1E. PLCβ1^+/+^ and PLCβ1^-/-^ mice were trained for either 3 days or 4 days (1S*3Days, 1S*4Days) for olfactory discrimination. Mice in Devalue group received sugar→nausea pairing. Maintain group received unpaired sugar and nausea. In 1S*3Days case, both genotypes formed a taste aversion (Fig 5A). When compared with Maintain groups, both PLCβ1^+/+^ and PLCβ1^-/-^ Devalue groups formed a significant aversion to sucrose pellets. ANOVA [(genotype—PLCβ1^+/+^, PLCβ1^-/-^) X (group—Devalue, Maintain)] for aversion index (AI) found a main effect of group [*F*(1,25) = 44.73, *p* = .000], but no effect of or interaction with genotype (*p*s > .5). Conditioned responding (CR) tests were conducted before (Pre-nausea) and after (Post-nausea) the CTA procedure (Fig 5C). In Pre-nausea CR session, all groups showed sucrose-seeking behavior in response to +odor. However, in Post-nausea CR session, Devalue groups showed significantly smaller CR compared with Maintain groups in both genotypes. Post-nausea CR of Devalue groups was significantly smaller than Pre-nausea CR in both genotypes. ANOVA [(genotype—PLCβ1^+/+^, PLCβ1^-/-^) X (group—Devalue, Maintain) X (nausea–Pre-nausea, Post-nausea)] for CR found significant main effects of nausea [*F*(1,25) = 15.73, *p* = .001] and group [*F*(1,25) = 4.56, *p* = .043], and a significant nausea X group interaction [*F*(1,25) = 16.97, *p* = .000], but no effect of nor any interaction with genotype (*p*s > .5). Post hoc comparisons found significant differences in Post-nausea CR between Devalue and Maintain groups (*p*s < .05), and between Pre-nausea and Post-nausea CRs of Devalue groups, in both genotypes (*p*s < .05) (within-subject paired t-test: *p*s < .05). These results show that in 1S*3Days case CR was reduced following devaluation of reward in both genotypes.

The CTA results of 1S*4Days case (Fig 5B) were similar to those of 1S*3Days (Fig 5A). ANOVA [(genotype—PLCβ1^+/+^, PLCβ1^-/-^) X (group—Devalue, Maintain)] for AI found a main effect of group [*F*(1,27) = 42.63, *p* = .000], but no effect of or interaction with genotype (*p*s > .1). CR experiments (Fig 5D) also showed similar results to those of the 1S*3Days case (Fig 5C). ANOVA [(genotype—PLCβ1^+/+^, PLCβ1^-/-^) X (group—Devalue, Maintain) X (nausea—Pre-nausea, Post-nausea)] for CR found significant main effects of nausea [*F*(1,27) = 10,84 *p* = .003] and group [*F*(1,27) = 6.92, *p* = .014], and a significant nausea X group interaction [*F*(1,27) = 11.51, *p* = .002], but no effect of nor any interaction with genotype (*p*s > .5). Post hoc comparisons found significant differences in Post-nausea CR between Devalue and Maintain groups (*p*s < .05), and between Pre-nausea and Post-nausea CRs of Devalue groups, in both genotypes (*p*s < .05) (within-subject paired t-test: *p*s < .005). These results and those of the 1S*3Days case above show that CR was sensitive to the current motivational value of the sugar reward in both genotypes, regardless of the amount of +odor→sugar training. In 1S*4Days case, therefore, the inability of PLCβ1^+/+^ mice to perform mediated learning cannot be explained by their initial olfactory learning being of S-R type.

## Discussion

The present study is the first to demonstrate both the normal and pathological courses of sensitivity to RMTA in mice. With a minimal amount of initial +odor→sugar associative training (1S*3Days), both wild-type and PLCβ1^-/-^ mice established an aversion to the sugar reward when the odor cue predicting sugar (+odor) was paired with nausea. With an extended initial training (1S*4Days), however, only PLCβ1^-/-^ mice could form a RMTA. This abnormal, persistent RMTA over the course of initial conditioning observed in PLCβ1^-/-^ mice shows that they cannot distinguish real sugar from the associatively-activated representation of sugar at a stage in conditioning where wild-type mice normally can differentiate the two. In reward devaluation task, both genotypes responded to +odor with reduced CRs when they had previously received a direct sugar→nausea pairing, regardless of the amount of initial +odor→sugar training. In the 1S*4Days case, therefore, the failed RMTA exhibited by wild-type mice cannot be attributed to their initial olfactory learning being a S-R association. Despite the fact that the initial conditioning was formed in S-S type which is supposed to support the mediated learning, RMTA did not happen in wild-type mice with 1S*4Days training condition. In other words, even though the odor CS evoked US representations in both genotypes with both initial training conditions, the US representation evoked in wild-type mice with 1S*4Days condition could not support mediated learning. This suggests that the US representation in wild-type mice with the 1S*4Days condition is of a nature different from that with 1S*3Days condition. It is likely that, with extended training, the US representation lost the ability to enter into a new learning, even though it still maintained some other aspects that can mediate proper conditioned responding. There seems to be a hypothetical normal transition of the nature of CS-evoked US representation as the wild-type mice proceed to the “extended” 4-day training, which does not happen in PLCβ1^-/-^ mice.

Identifying the neural dysfunction related to impaired reality testing calls for application of this behavioral tool to a variety of genetic mouse models of schizophrenia and related disorders. The previous study with NVHL rats demonstrated an “enhanced” RMTA rather than differential effects of initial training amount on sensitivity to RMTA, due to the difficulty in deciding the minimal training condition that enables RMTA in normal adult rats [16]. The present study with PLCβ1^-/-^ mice has shown, using a well-defined minimal amount of odor-taste training, the first genetic mouse model of schizophrenia that displays impaired reality testing with an abnormal phenomenon of “persistent” RMTA. This study not only supports the RMTA procedure as an experimental tool for assessing reality testing function, but also suggest that its principle can be applied in general terms to a variety of animal models by finding a paradigm suitable for each specific case (e.g. modality of conditioned cue, manipulation of amount of initial training, etc.).

Typical antipsychotics such as D2 dopamine antagonists (e.g. haloperidol) relieve the positive symptoms of schizophrenia, including hallucinations. For the predictive validity of this animal model, we will examine whether these antipsychotics could reverse the persistence of RMTA in PLCβ1^-/-^ mice. We will also perform similar experiments (e.g. effects of the amount of initial training in both genotypes) with another mediated learning paradigm, mediated “extinction” of taste aversion [29], which will help to further support mediated learning paradigms as an animal behavioral model tool for impaired reality testing. With Pavlovian conditioning paradigms designed for humans, translational approaches may look for psychological test outcomes in schizophrenia patients that are comparable to the abnormal course of sensitivity to RMTA in animal model. CS-evoked brain response can be monitored using event-related functional magnetic resonance imaging (efMRI) [30]. Then it would be worthwhile to examine whether differential patterns of CS-evoked efMRI in the course of Pavlovian conditioning (e.g. minimal and extended training) can be found in control subjects versus schizophrenia patients.

Rat studies using tone-sugar [31] or odor-sugar pairing [24] found learning-dependent activation of neurons, as measured by immediate early gene (IEG) expression, in gustatory cortex (GC) by CS. A rat study by Dwyer and Killcross with context-taste conditioning showed that basolateral amygdala (BLA) is necessary for mediated taste aversion learning [32], and it was suggested that BLA may be involved in encoding perceptual information in GC while CS evokes a representation of US. Given the hypothesis that associatively-activated representation may contain sensory-perceptual features to support mediated learning, it could be expected that BLA lesion might also interfere with normal activation of “sensory” component of CS-evoked taste representation. However, a study on odor CS-activated taste representation using IEGs expression in GC showed that BLA lesion did not disrupt the odor-evoked sensory representation of sugar [24]. Consistent with this result, another study using odor-sugar association done in the same laboratory has shown that odor-mediated taste aversion learning does not require BLA but dorsal hippocampus [25]. It seems that the elements of neural systems involved in mediated learning and/or sensory encoding of CS-evoked representation can differ depending on the modality of CS. It would be worthwhile to examine whether dorsal hippocampus is required for RMTA with odor CS also in mice, and if so, to determine whether and how it is involved in the sensory encoding of CS-evoked representation on the GC. Future studies with this RMTA paradigm using functional histology, in vivo neurophysiology, and in vivo two-photon calcium imaging [33], will help to find neural substrates for the different natures of associatively-activated event representations, so as to address the hypothetical transition in the nature of CS-evoked representation over the course of the initial conditioning in anatomical and physiological terms. For example, we should analyze patterns of CS-evoked neural activation (e.g. Fos expression, IEGs in situ hybridization) in GC for 1S*3Days and 1S*4Days conditions, such as degrees of overlap with the neural activity evoked by actual sugar US, in PLCβ1^-/-^ and wild-type mice, to see whether we can detect the hypothetical difference between the contents of the associative representations for 1S*3Days and 1S*4Days conditions (e.g., only in wild-type but not in PLCβ1^-/-^ mice).

The persistent RMTA in PLCβ1^-/-^ mice suggests that PLCβ1 is necessary for the normal course of sensitivity to RMTA and/or the normal shift in the nature of associatively-activated representation. By asking how PLCβ1 may be involved in the hypothetical normal shift in the nature of associatively-activated event representation, we will seek to investigate the neural mechanisms underlying the transition between these different types of CS-activated representation, using molecular tools such as conditional knock-out or local knock-down of this gene in the brain areas. This study utilizing RMTA paradigm would contribute greatly to research on neural basis of impaired reality testing, related mental illness symptoms, and development of novel therapeutics, as well as to better understanding of associatively-activated event representation in the field of learning theory.
